# Supplementary material for: Elucidation of the Relationship between Intrinsic Viscosity and Molecular Weight of Cellulose Dissolved in Tetra-N-Butyl Ammonium Hydroxide/Dimethyl Sulfoxide
Source: Polymers (Basel). 2019 Oct 1;11(10):1605. doi: 10.3390/polym11101605 (PMC6836168; doi:10.3390/polym11101605)
Supplement: Supplementary file 1 [file polymers-11-01605-s001.pdf]

polymers

Supporting Information

Elucidation of the Relationship between Intrinsic Viscosity and Molecular Weight of Cellulose Dissolved in Tetra-N-Butyl Ammonium Hydroxide/Dimethyl Sulfoxide

Daqin Bu, Xiangzhou Hu, Zhijie Yang, Xue Yang, Wei Wei, Man Jiang*, Zuowan Zhou, Ahsan Zaman

Key Laboratory of Advanced Technologies of Materials (Ministry of Education), School of Materials Science and Engineering, Southwest Jiaotong University, 610031, Chengdu, P.R. China

* Correspondence: jiangman1021@swjtu.edu.cn; Tel.: +86-28-87601980

1. Viscosity Analysis of the Cellulose in Various Solution

Cellulose rapidly dissolved in the THDS system to form a transparent uniform solution, as per Video S1.

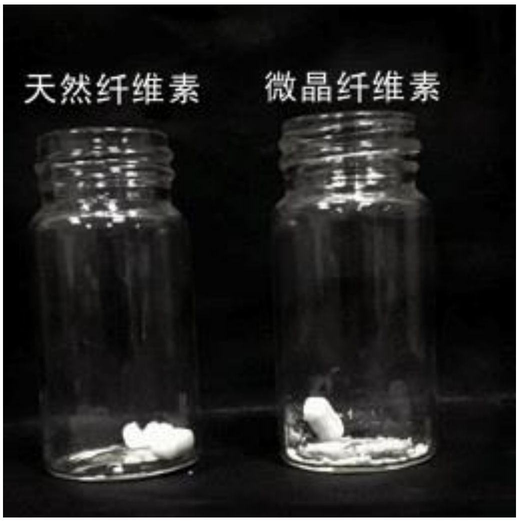

Video S1. Rapid dissolution of cellulose.

Pictures of the cellulose/Cuoxam solution, the cellulose/CED solution, and the cellulose/THDS solution are shown in Figure S1. It could be seen that the cellulose/THDS solution was transparent and bright in color, the cellulose/ Cuoxam solution was blue, and the cellulose/CED solution was the darkest in color. The cellulose/THDS solution could be dissolved in just a few minutes, making it easy to prepare and saving time, and the transparent solution was easy to observe.

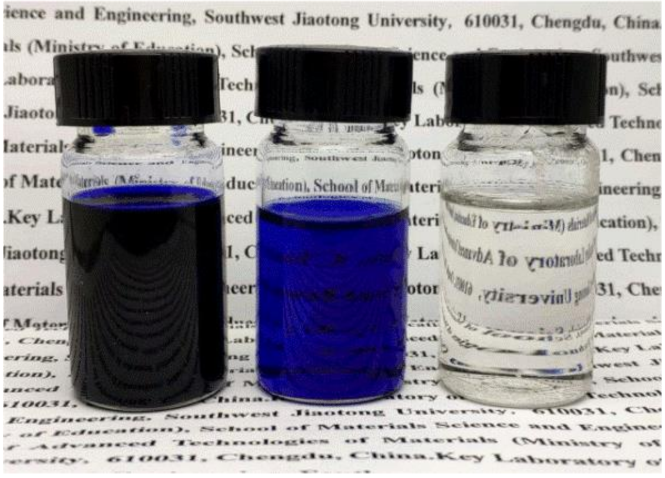

Figure S1. Photograph of the cellulose/CED solution (left), cellulose/Cuoxam solution (middle), and the cellulose/THDS solution (right).

2. The Elucidation of the Mark–Houwink Equation for the Cellulose/THDS Solution

The value of the parameter α in the Mark–Houwink equation has been reported.

Table S1. Parameters for the Mark–Houwink equation of cellulose dissolved in various solvents.

Solvent	Temperature (℃)	K ($cm^3 \cdot g^{-1}$)	α	Method for DP Determination	Reference
DMAc/LiCl	30	0.054 [b]	1.19	Light scattering	Dupont 2003
BmimAc/DMSO	25	2.5×10^{-4} [a]	0.83	Viscosity method	Liu et al. 2016
PF/DMSO	30	3.01 [b]	0.81	Light scattering	He and Wang 2000
NaOH/urea	25	2.45×10^{-2} [a]	0.815	Light scattering	Shi et al. 2018
LiOH	25	2.78×10^{-2} [a]	0.79	Light scattering	Cai et al. 2006
Cuoxam	25	0.8 [b]	0.81	Light scattering	He and Wang 2000
CED	25	17.0 [b]	0.80	Osmotic Pressure	Liu 1985
Cadoxen	25	1.8 [b]	0.77	Light scattering	He and Wang 2000
NH3/NH4SCN	25	0.862 [b]	0.95	-	Kasaai 2002
THDS	25	0.24 [b]	1.21	Viscosity method	This work

[a] $[\eta] = K \times M^{\alpha}$, [b] $[\eta] = K \times DP^{\alpha}$.

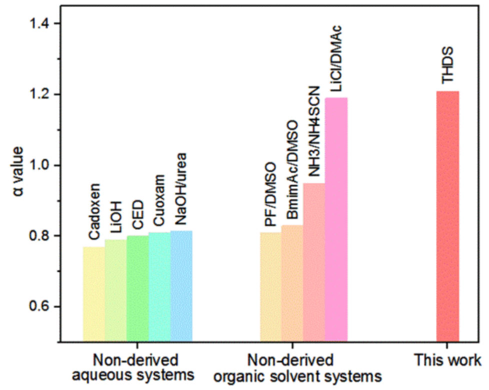

Figure S2. Comparison of the α values in the Mark–Houwink equation of different cellulose solvents.

3. FT-IR Characterization of Cellulose Acetate Samples

Fourier-transform infrared (FT-IR) spectra were recorded on a Nicolet 6700 (Thermo Scientific Inc., Waltham, MA, USA) with the KBr-technique, with a spectral resolution of 2 cm^{-1} in the range from 4000 to 400 cm^{-1} at room temperature. In the sample preparation and measurement process, infrared light was used to bake in order to remove moisture.

The spectra cellulose acetate samples CA1–CA5 (Figure S3) provide a clear evidence of acetylation by showing the presence of some important peaks at 3400 cm^{-1} for OH stretching, 1750 cm^{-1} for C=O stretching in ester, 1240 cm^{-1} for C–O–C stretching, and 1380 cm^{-1} for CH_3 bending.

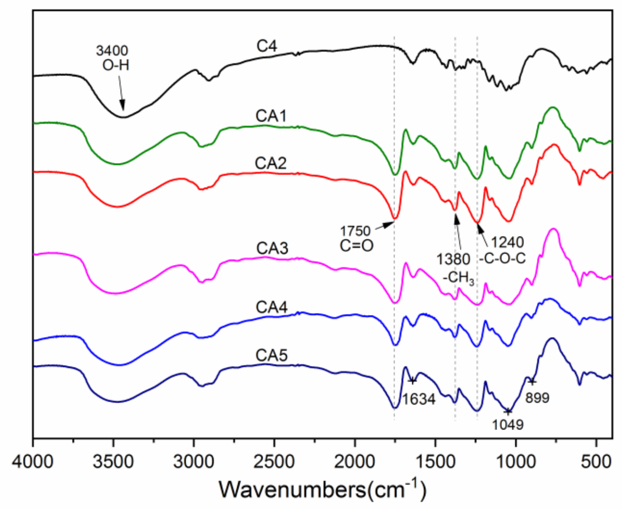

Figure S3. FT-IR spectra of samples (cellulose C4 and cellulose acetate samples CA1–CA5).

4. ^{1}H NMR Characterization of Cellulose Acetate Samples

^{1}H NMR spectra were recorded in DMSO-d_6 (50 mg/mL) with a Bruker AMX 400 spectrometer running at 400 MHz, at 30 °C, with 16 scans. The DS of acetylated cellulose could be calculated by ^{1}H NMR spectroscopy according to the following equation:

$$\mathrm{DS} = \frac{n_{acetyl} \times I_{acetyl}}{n_{AGU} \times I_{AGU}}, \quad (1)$$

where I_{acetyl} is the integral of methyl protons of acetyl groups, I_{AGU} is the integral of all protons of the anhydroglucose unit, n_{acetyl} is the number of protons in the glucose ring, and n_{AGU} is the number of methyl proton in acetate groups.

A ^{1}HNMR spectrum of cellulose acetate samples is shown in Figure S4. The DS is readily calculated from the ratio of the spectroscopic integral of methyl protons of acetyl groups (δ = 1.7–2.2 ppm) and the protons of the anhydroglucose unit (δ = 3.5–6.0 ppm).

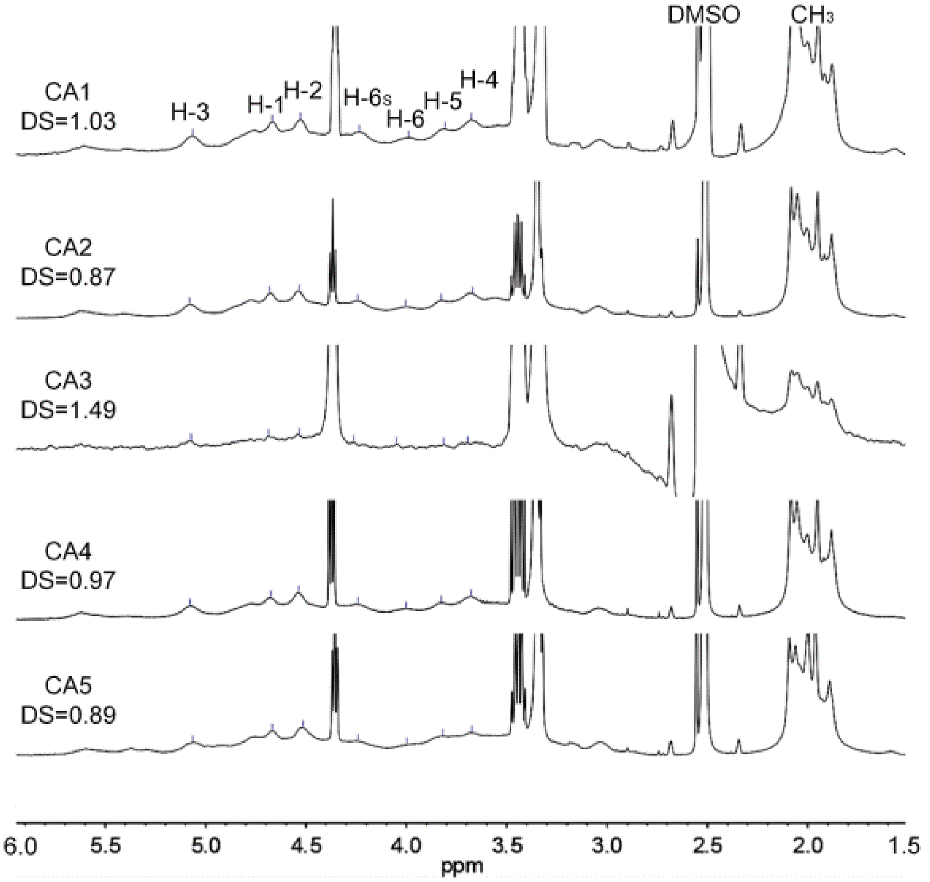

Figure S4. ^{1}H NMR spectra of cellulose acetate samples CA1–CA5 in DMSO-d_6 recorded.
